# A Review of You Only Look Once Algorithms in Animal Phenotyping Applications

**DOI:** 10.3390/ani15081126

**Published:** 2025-04-13

**Authors:** Guangbo Li, Rui Jian, Xie Jun, Guolong Shi

**Affiliations:** 1 College of Command and Control Engineering, Army Engineering University of the People’s Liberation Army, Nanjing 210007, China; liguangbo@hblgxy.edu.cn; 2 College of Electronic and Information Engineering, Huaibei Institute of Technology, Huaibei 235000, China; 3 School of Information and Artificial Intelligence, Anhui Agricultural University, Hefei 230036, China

**Keywords:** animal phenotype, YOLO-series algorithms, dataset, deep learning

## Abstract

Animal phenotyping recognition is a pivotal component of precision livestock management, holding significant importance for intelligent farming practices and animal welfare assurance. In recent years, with the rapid advancement of deep learning technologies, the YOLO algorithm—as the pioneering single-stage detection framework—has revolutionized the field of object detection through its efficient and rapid approach and has been widely applied across various agricultural domains. This review focuses on animal phenotyping as the research target structured around four key aspects: (1) the evolution of YOLO algorithms, (2) datasets and preprocessing methodologies, (3) application domains of YOLO algorithms, and (4) future directions. This paper aims to offer readers fresh perspectives and insights into animal phenotyping research.

## Simple Summary

As the first one-stage detection algorithm in the field of object detection, the YOLO algorithm has opened up a new direction in the field of object detection with its simple and fast detection method and is widely used in various fields of agriculture. This article mainly takes animal phenotypes as the research target and elaborates on the YOLO series of algorithms from the following aspects: (1) the development of the YOLO algorithm; (2) the dataset and preprocessing; (3) the application fields of the YOLO algorithm; and (4) future development directions. Thus, it provides readers with new understandings and considerations in the field of animal phenotypes.

## 1. Introduction

Animal phenotyping research, as an interdisciplinary frontier integrating modern animal husbandry and computer vision, originated from the quantitative demands of traditional morphometrics in characterizing animal physiological traits [1]. Initial studies primarily relied on manual measurements and visual assessments, which were characterized by low efficiency and high subjectivity. The late 20th century witnessed the introduction of semi-automated methods [2], such as threshold segmentation [3] and edge detection [4], through advancements in image processing technology. However, these approaches suffered from insufficient robustness in addressing dynamic interferences in complex farming environments. The breakthroughs in deep learning technologies after 2012 catalyzed the rapid evolution of object detection algorithms. Notably, two-stage algorithms like Faster R-CNN [5], through the “region proposal-refined detection” workflow, significantly enhanced detection accuracy. Nevertheless, their high computational costs and low real-time performance constrained large-scale agricultural applications. Against this backdrop, the YOLO (You Only Look Once) series [6,7,8,9,10,11,12,13] of algorithms emerged, achieving a balanced optimization of speed and accuracy through end-to-end single-prediction mechanisms. These algorithms have become the technological cornerstone for intelligent animal phenotyping research. From YOLOv1 [6] to YOLOv9 [13], iterative advancements have been made, including multi-scale feature fusion, attention mechanisms, and lightweight designs. These innovations have reduced hardware deployment barriers and provided a new paradigm for non-invasive, high-dynamic phenotypic analysis.

Animal phenotyping research holds profound theoretical and practical significance. Theoretically, phenotypic data serves as a critical bridge for elucidating the interaction mechanisms between animal traits and environmental factors, providing essential evidence for genetic breeding and evolutionary biology [14]. Practically, precise phenotypic analysis can optimize feed efficiency, enable the early detection of abnormal behaviors and diseases, and improve animal welfare.

However, traditional methods face significant challenges: manual measurements and RFID tagging [15] are invasive, costly, and environmentally adaptive. Conventional image processing techniques like threshold segmentation are susceptible to lighting variations and occlusions [3]. Early deep learning models such as Mask R-CNN [16] suffer from high computational complexity, limiting their practicality in resource-constrained farms. The YOLO series addresses these bottlenecks through three key innovations: (1) lightweight architecture: YOLOv5’s CSPDarknet backbone supports real-time inference on edge devices at speeds up to 100 frames per second [17]; (2) adaptive data augmentation and multi-task scalability: the literature [18] integrates keypoint detection and instance segmentation for integrated phenotypic parameter extraction; and (3) enhanced scalability: the literature [19] improves cross-scenario robustness through transfer learning.

Despite significant progress, challenges remain: (1) lack of dataset standardization: existing animal phenotyping datasets suffer from small scale, inconsistent annotation standards, and poor cross-species generalizability; (2) lagging multimodal fusion: current research primarily relies on RGB imagery, failing to integrate infrared, depth sensing, and other multimodal data; and (3) computational constraints: real-time multi-target detection on edge devices remains computationally intensive. Recent reviews [20,21,22,23,24] have addressed YOLO algorithms, but few focus specifically on animal phenotyping. This paper systematically examines four aspects, as follows:

(1) The architectural evolution of YOLO algorithms and their adaptability to phenotypic tasks; (2) construction logic, annotation protocols, and preprocessing techniques for animal phenotyping datasets; (3) practical efficacy of YOLO in body measurement, individual identification, behavior monitoring, and biomass estimation; and (4) future challenges and emerging directions.

## 2. Materials and Methods

### 2.1. Development of the Target Detection of YOLO Family Algorithms

**Figure 1:**
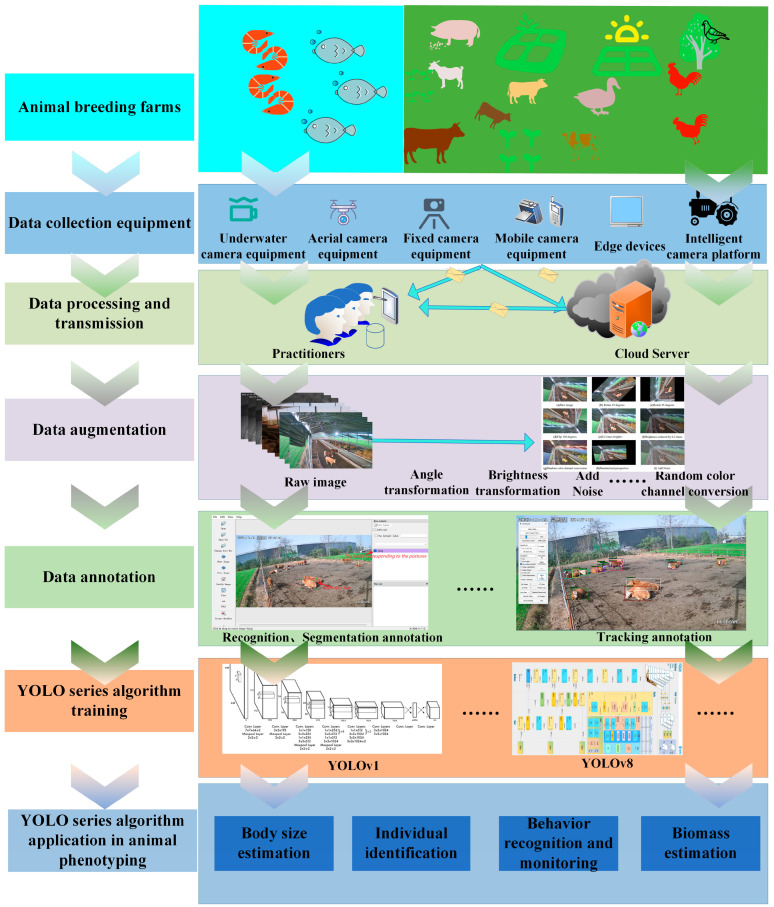
Intelligent applications of YOLO-series algorithms in animal phenotyping.(“星期日” is Sunday. “星期四” is Thursday.)

The application workflow of YOLO algorithms in intelligent animal phenotyping is illustrated in Figure 1.

As shown in Figure 1, the intelligent application workflow of YOLO-series algorithms in animal phenotyping comprises six critical steps:

(1) Data Collection: Data collection is conducted in livestock farms using flexible equipment selection based on environmental conditions, including underwater cameras, fixed cameras, handheld devices, drones, and edge devices.
(2) Data Transmission: Collected data are stored locally or in cloud platforms to facilitate data retrieval and processing.
(3) Data Augmentation: Data enhancement techniques such as rotation, flipping, brightness adjustment, random color channel permutations, random perspective transformations, and noise addition are applied to expand dataset diversity.
(4) Data Labeling: Data are labeled according to specific application scenarios.
(5) Model Training: Labeled datasets are input into appropriate YOLO-series algorithms for model training, optimizing detection accuracy and generalization capabilities.
(6) YOLO-Based Phenotyping Applications: The trained YOLO models are deployed for animal body size estimation, individual identification, behavioral recognition and monitoring, and biomass estimation, enabling intelligent livestock management and sustainable farming practices.

**Figure 2:**
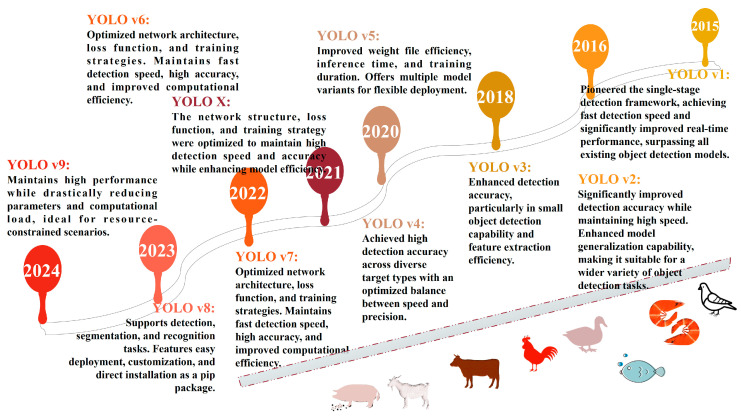
Development flowchart of YOLO-series algorithms.

As illustrated in Figure 2, the YOLO series has undergone substantial iterative refinement, with increasing adoption in animal phenotyping research. While each version exhibits distinct mechanisms, advantages, and limitations (summarized in Table 1), their specialized strengths enable tailored adaptations to specific phenotypic tasks.

#### 2.1.1. YOLOv1

As a single-stage algorithm, YOLOv1 [6] processes input images through three sequential steps: First, the input image is divided into equal-sized grids to partition the visual space. Next, the algorithm predicts bounding boxes (specifying center coordinates, height, width, and confidence scores) for objects within each grid while simultaneously assigning confidence scores to indicate the likelihood of each class being present. Finally, non-maximum suppression (NMS) is applied to eliminate redundant or overlapping bounding boxes, ensuring only the most plausible predictions remain.

YOLOv1 demonstrated advantages in speed (FPS), recall rate, and generalizability compared to traditional methods. However, it exhibited limitations in precision, localization accuracy, and small-object detection performance. Despite these drawbacks, YOLOv1 laid the foundational architecture for subsequent iterations, enabling advancements in network design and loss optimization.

#### 2.1.2. YOLOv2

YOLOv2 [7] (also known as YOLO9000), which was built upon YOLOv1, introduced a new DarkNet-19 backbone network and implemented four critical enhancements:

(1) Batch normalization: Integrated after convolutional layers to accelerate training, improve stability, and enhance generalization.
(2) Resolution scaling: Fixed input resolution at 448 × 448 pixels to preserve spatial details for higher accuracy.
(3) Anchor box mechanism: Borrowed from Faster R-CNN, this approach employs predefined anchor boxes to optimize prediction speed and accuracy.
(4) K-Means clustering [25]: Applied to cluster training data bounding boxes, ensuring anchor box dimensions align with target sizes.

YOLOv2 balanced speed and precision. The literature [26] reported average accuracies of 93.94% and 97.06% for two fish species using YOLOv2 in continuous-frame classification tasks.

#### 2.1.3. YOLOv3

YOLOv3 [8] represents a significant advancement in the YOLO series, featuring the following improvements:

(1) Network architecture enhancement: Upgraded from DarkNet-19 to DarkNet-53, deepening the network depth and improving performance. This strengthened the model’s feature extraction capabilities, enhancing detection accuracy.
(2) Introduction of FPN structure: Integrated the Feature Pyramid Network (FPN) to fuse multi-scale feature information, bolstering the model’s ability to detect objects of varying sizes. Through multi-scale predictions, it demonstrated particularly enhanced detection sensitivity for small objects. Based on this improvement, the YOLO-series network is divided into four components: input network, backbone network, neck, and detection head.

YOLOv3 maintains high detection accuracy while achieving fast inference speed, meeting real-time detection requirements. The literature [27] reported 96% average accuracy for cattle face recognition using YOLOv3 combined with infrared thermography.

#### 2.1.4. YOLOv4

YOLOv4 [9], introduced in 2020, represents another significant advancement in the YOLO series, featuring the following improvements:

(1) Input Enhancements: Implemented Mosaic data augmentation, Cross Mini-Batch Normalization (CMBN), and Self-Adversarial Training (SAT) to enhance dataset diversity and model robustness.
(2) Backbone Optimization: Replaced Darknet-53 with CSPDarknet53, improving learning capacity while reducing computational bottlenecks and memory costs.
(3) Neck Structure: Incorporated Spatial Pyramid Pooling (SPP) and FPN + PAN architectures to better fuse multi-scale features and expand the receptive field, boosting detection performance.

YOLOv4 maintained high detection speed while achieving improved accuracy across diverse target types, demonstrating a superior balance between speed and precision. However, its complex architecture poses challenges in training and deployment, requiring substantial computational resources. The literature [28] utilized YOLOv4 with a GhostNet module to refine feature fusion networks, achieving 96.7% average accuracy and 28 FPS for goat face recognition.

#### 2.1.5. YOLOv5

YOLOv5 represents a new generation of algorithms in the YOLO series, incorporating the essence of prior iterations with significant improvements in weight files, inference time, and training efficiency compared to YOLOv3 and YOLOv4. YOLOv5 is structured into four primary components: input (input terminal), backbone (main network), neck (neck network), and prediction (output terminal).

The literature [29] demonstrated the application of YOLOv5 by integrating MobileNetV3 and attention mechanisms, achieving a porcine body estimation model with reduced parameter size (10.2 M) and an error rate below 2%.

#### 2.1.6. YOLOX

YOLOX [10], as an advanced one-stage target detection algorithm, achieves significant performance improvements through multiple innovative mechanisms. First, it adopts an anchor-free design that abandons traditional anchor-box-based detection by directly predicting object center coordinates and dimensions, thereby simplifying the model architecture and reducing dependency on hyperparameters. Second, it employs a decoupled-head strategy to separate classification and bounding box regression tasks into independent branches, enabling specialized optimization of feature representations for enhanced detection accuracy. Third, it introduces SimOTA labeling, a dynamic sample assignment method based on optimal transport theory, which resolves sample conflicts in dense-target scenarios by adaptively allocating positive/negative samples according to prediction–realization matching costs. Additionally, YOLOX integrates Mosaic and MixUp data augmentation through multi-image stitching and blending to strengthen environmental generalization while utilizing an end-to-end optimization framework for efficient feature mapping and parameter learning from input to output.

The literature [30] demonstrated the effectiveness of YOLOX by incorporating normalized attention mechanisms, optimized loss functions, and lightweight spatial pyramid structures, achieving 97.57% average accuracy for group pig behavior recognition.

#### 2.1.7. YOLOv6

YOLOv6 [11], developed by Meituan (Beijing, China), introduced a series of optimizations and adjustments to network architecture, loss functions, and training strategies, achieving moderate improvements in detection accuracy. By redesigning the decoupled-head structure with a hybrid channel strategy, it significantly reduced computational resource consumption while enhancing operational efficiency. YOLOv6 is suitable for animal phenotyping recognition in edge scenarios of livestock farms.

#### 2.1.8. YOLOv7

YOLOv7 [12] further improved the YOLO-series network through the following enhancements:

(1) Network architecture optimization: Introduced more efficient convolutional layers, attention mechanisms, and path aggregation networks to enhance performance and speed.
(2) Transformer module integration: Deployed Transformer-based attention mechanisms to improve the model’s understanding of image features, boosting recognition accuracy in complex scenarios.
(3) Feature extractor refinement: Utilized deeper and more complex network structures to extract advanced features, enabling better image content interpretation and detection precision.
(4) Input/output optimization: Implemented various optimization techniques in input and output network components to achieve a balanced trade-off between detection speed and accuracy, providing a robust foundation for diverse applications.

The literature [31] demonstrated the application of YOLOv7 by incorporating an EMobileNet backbone and horizontal–vertical attention mechanism (HVAM), achieving a porcine face recognition model with 0.97 M parameters, 99.34% average accuracy, and 120 FPS.

#### 2.1.9. YOLOv8

YOLOv8 introduced innovations in backbone network, detection head, and loss function design, achieving improved detection accuracy compared to YOLOv5, YOLOv6, and YOLOv7. The algorithm replaced C3 blocks with C2f structures to enhance gradient flow dynamics while optimizing channel configurations for multi-scale adaptability. Its output network employs a decoupled-head architecture to separately predict object location and class features.

The literature [32] demonstrated the application of YOLOv8 by integrating SENet attention mechanisms and WIoU v3 loss functions, achieving 94.4% average accuracy for duck flock behavior recognition, with model size reduced by 2.8 MB (8.7% parameter reduction).

#### 2.1.10. YOLOv9

YOLOv9, building upon YOLOv7, introduced Programmable Gradient Information (PGI) and the General Efficient Layer Aggregation Network (GELAN) to enhance performance efficiency. By reducing parameter count and computational demands while maintaining high accuracy, YOLOv9 demonstrates superior suitability for resource-constrained devices. Its architectural strengths enable broad applications in animal phenotyping, including body size estimation, individual identification, behavioral monitoring, and biomass assessment.

### 2.2. Evaluation Metrics

This study employs commonly used evaluation metrics for animal phenotyping recognition algorithms. Precision (*P*) represents the proportion of correctly predicted positive samples, while Recall (*R*) denotes the ratio of true positive samples correctly identified. Average Precision (*AP*) is calculated for individual classes, and mean Average Precision (mAP) is the average of AP values across all classes. Two variants are reported based on Intersection over Union (IoU) thresholds: mAP50 (IoU = 0.5) and mAP50:95 (IoU = 0.5–0.95 with 0.05 increments). True Positives (TPs) count correctly detected targets, False Positives (FPs) represent erroneous detections, and False Negatives (FNs) indicate missed targets. Frame rate (FPS) measures processing speed in frames per second. Floating Point Operations (FLOPs) evaluate computational complexity, reflecting the algorithm’s hardware resource demands for single-forward pass inference.

(1) $$Precision=\frac{TP}{TP+FP}$$

(2) $$Recall=\frac{TP}{TP+FN}$$

(3) $$AP=\int_{0}^{1}P(R)dR$$

The experimental results of YOLO-series algorithms, as shown in Table 2 and Table 3, demonstrate significant improvements in metrics such as mAP and FPS across algorithm iterations. These advancements further validate the feasibility of YOLO-based solutions in animal phenotyping applications, providing technological support for intelligent livestock farming and management.

### 2.3. Datasets and Preprocessing

Datasets form the foundation for training and validating YOLO algorithms, directly influencing model performance and generalization capabilities. The diversity of datasets—encompassing variations in species, environments, and lighting conditions—is critical for ensuring model robustness. Well-curated datasets provide accurate data features for object detection models, enhancing detection efficacy. Datasets are broadly categorized into public datasets and custom datasets. Public datasets play a vital role in benchmarking model performance, reducing data acquisition costs, fostering algorithmic research, and enabling pretrained models. Researchers leverage public datasets to compare algorithms under standardized conditions, optimize methodologies, and avoid the high costs of data collection and annotation. Additionally, public datasets are widely used for pretraining models to improve task-specific performance. Custom datasets, on the other hand, are tailored by researchers to address specific requirements, enhance model specialization, overcome data scarcity, and advance domain-specific applications.

#### 2.3.1. Public and Custom Datasets

The YOLO-series algorithms, from YOLOv1 to YOLOv9, have relied on public datasets for training and validation. The characteristics of public datasets—such as diversity, scale, and annotation quality—play a critical role in enhancing YOLO algorithm performance.

**Figure 3:**
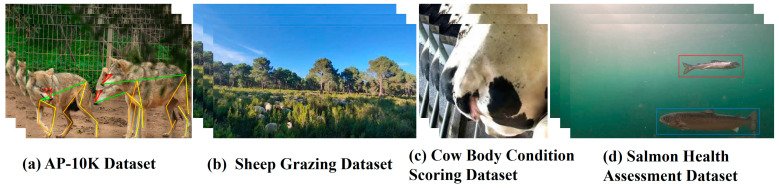
Animal phenotyping data visualization examples.

As shown in Table 4, public datasets have evolved with advancements in hardware and software technologies, increasingly featuring diverse target categories, large sample sizes per category, and high-resolution images. Animal phenotyping public datasets further focus on specific challenges in intelligent livestock farming. For example, the AP-10 K Animal Pose Estimation Dataset [33] (Figure 3a) concentrates on animal posture recognition. The Sheep Grazing Dataset [34] (Figure 3b) addresses intelligent sheep grazing management. The Cattle Health Assessment Dataset [35] (Figure 3c) focuses on cattle body condition scoring to support livestock health monitoring. The Salmon Health Evaluation Dataset [36] (Figure 3d) targets salmon growth status analysis for sustainable aquaculture. These animal phenotyping public datasets provide standardized samples for solving domain-specific farming problems, establish benchmarks for comparing YOLO-based algorithms, and facilitate further advancements in animal phenotyping applications.

While public datasets save time and resources while providing standardization, their limitations become evident as technology advances and application scenarios expand. Consequently, YOLO algorithms in animal phenotyping research increasingly rely on proprietary datasets. These datasets offer broader applicability, enabling the creation of domain-specific data for animal facial features, body postures, and behavioral patterns in livestock farming.

In animal phenotyping applications, experts utilize proprietary datasets to address specific challenges through domain-specific data construction. For instance, Guo Yangyang et al. [37] developed sheep facial datasets to enable individual identification and precision breeding in goats, while Huang Xiaoping et al. [38] created cattle body condition datasets to standardize commercial body scoring systems for livestock health monitoring. Similarly, Guo Jianjun et al. [39] designed pigeon behavior datasets to facilitate behavioral pattern recognition for scientific breeding optimization. These tailored datasets directly align with practical farming needs, demonstrating how proprietary data collection addresses niche challenges such as species-specific identification, health assessment, and behavioral analysis in animal husbandry.

As shown in Table 4 and Table 5, proprietary animal phenotyping datasets exhibit the following advantages over public datasets:

(1) Sample Focus: Animal phenotyping self-built datasets are tailored to address specific production challenges, such as sheep individual identification, through dedicated facial datasets [37].
(2) Small Data Volume: These datasets are designed for single-task optimization, resulting in compact data size, low resource consumption, and ease of deployment.
(3) Convenient Data Collection: Their focused scope and limited scale enable straightforward collection using cameras in real-world farming environments.

There are also limitations to self-built datasets:

(1) Poor Scalability: The closed nature of animal phenotyping data restricts sample diversification, limiting their applicability to related problems.
(2) Low Reproducibility Assurance: Insufficient documentation and transparency in dataset construction hinder researchers’ ability to reproduce or improve upon them for algorithmic advancements.

#### 2.3.2. Annotation Methods

Data annotation is a critical step in building high-quality datasets for animal phenotyping. As shown in Table 6, different annotation methods serve distinct research tasks and application scenarios.

**Figure 4:**
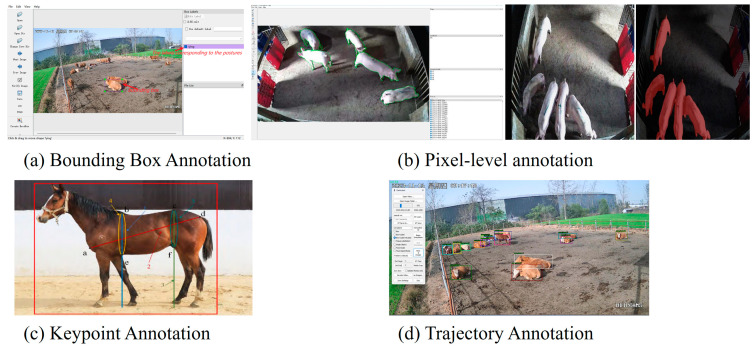
Schematic diagram of animal data annotation methods for YOLO-series algorithms.

Bounding box annotation [40] (Figure 4a) involves labeling target animals with rectangular boxes to define their positions and ranges. This method is widely used for individual detection, counting statistics, and target tracking. The literature [40] utilized the LabelImg software tool (https://github.com/tzutalin/labelImg (accessed on 20 January 2025)) to annotate cattle postures, fulfilling sample requirements for posture recognition tasks.

Semantic segmentation annotation [28] (Figure 4b) assigns category labels to each pixel, enabling contour extraction, background separation, and scene understanding. This approach is particularly effective for multi-animal scene analysis and phenotypic feature quantification. The literature [28] applied the Labelme software(4.5.13) for the pixel-level annotation of pig bodies, providing data samples for group segmentation.

Keypoint annotation [18] (Figure 4c) labels critical body parts (e.g., head, limbs, joints) of animals. This method supports posture estimation, behavior recognition, and phenotypic measurement by providing precise positional information for detecting subtle changes and dynamic processes. The literature [18] used Labelme (https://github.com/wkentaro/labelme (accessed on 20 January 2025)) to annotate equine measurement points, establishing a dataset for horse body size analysis.

Trajectory annotation [41] (Figure 4d) tracks the movement paths of animals in videos, facilitating behavioral analysis, target re-identification, and group behavior studies. The literature [41] employed Dark Label software (https://github.com/darkpgmr/DarkLabel/releases/download/darklabel2.3-update2/DarkLabel2.3-update2.zip (accessed on 20 January 2025)) to annotate cattle trajectories, supporting data-driven livestock monitoring.

These diverse annotation methodologies cater to the multifaceted demands of animal phenotyping research, providing high-quality data support for model training and evaluation and thereby advancing the field of intelligent livestock farming and animal science.

## 3. Results

### 3.1. Analysis of YOLO Algorithms in Animal Phenotyping Applications

The YOLO-series algorithms demonstrate significant potential in animal phenotyping analysis due to their unique advantages. These algorithms have been applied across diverse animal categories, including livestock (cattle, pigs, sheep, poultry) and aquatic species (fish, shrimp, crabs), enabling multi-dimensional phenotyping analysis. Core tasks in this domain include object detection, keypoint monitoring, and instance segmentation.

Object detection employs end-to-end learning to identify animal targets in images or video frames, laying the foundation for analyzing abnormal behaviors or health conditions.

Keypoint detection captures anatomical landmarks such as body dimensions or skeletal structures, aiding experts in interpreting behavioral patterns and health status. For example, detecting keypoints like chicken claws or wings enables analysis of movement patterns and wellness.

Instance segmentation provides granular semantic information by precisely delineating animal body parts, supporting in-depth physiological studies.

Practical applications of YOLO algorithms in animal phenotyping encompass four primary areas:

(1) Body size estimation (assessing growth status via trunk or body length analysis to inform breeding decisions), (2) individual identification (recognizing unique features like facial or nasal characteristics), (3) behavior recognition and monitoring (continuous tracking of normal/abnormal activities for early intervention), and (4) biomass estimation (quantifying organ sizes or population counts for resource management). Specific research cases, including methodologies and outcomes, are summarized in Table 7.

#### 3.1.1. Body Size Estimation

Animal morphometric estimation is a technical methodology that quantitatively evaluates growth status and phenotypic characteristics through analysis of key morphological traits, including trunk dimensions, body length, withers height, and chest girth. Its primary objective is to provide scientific evidence for breeding management, genetic selection, and health monitoring. Standard morphometric parameters typically include body length (linear distance between cranial (head) and caudal (tail) endpoints), withers height (vertical measurement from ground to the highest dorsal point), chest girth (circumference of the thoracic cavity at its maximal expansion), and body weight estimation (derived via regression models constructed from morphometric parameters). Recent advancements integrate the YOLO algorithm with morphometric estimation to enhance automation and precision. YOLO’s real-time object detection capability enables the precise localization of animal individuals in images or videos. Subsequent keypoint detection or instance segmentation techniques extract morphological landmarks (e.g., head, dorsal line, tail base), which are then utilized to calculate morphometric parameters.

As demonstrated in the literature [45,46,47,49], YOLO-based frameworks trained on datasets acquired via handheld or fixed RGB cameras have established prediction systems for body length, weight, and volume. These systems facilitate intelligent livestock production through automated image analysis. Notably, a beef cattle weight estimation model using YOLOv8 achieved a mean recognition accuracy of 99% [47].

To optimize model efficiency without compromising accuracy, researchers have incorporated attention mechanisms, backbone network replacements, and layer pruning into YOLO architectures. For instance, the literature [43] applied sparse batch normalization (BN) layers and channel pruning to YOLOv5, achieving reductions of 86.10% in model size, 88.19% in parameter quantity, and 63.25% in FLOPs. Furthermore, integration with image segmentation techniques has expanded the scope of morphometric applications. The literature [50] combined YOLOv8 with the Roboflow algorithm to develop a holistic solution for goat weight estimation, while another study [51] employed YOLOv5 for sow ulcer localization and U-Net architecture for pixel-level lesion segmentation.

The integration of YOLO algorithms with body size estimation not only automates measurements but also delivers high accuracy and real-time processing for large-scale livestock farms. This approach supports non-contact monitoring, data-driven breeding decisions, and resource optimization, ultimately improving production efficiency and sustainability in animal husbandry.

#### 3.1.2. Individual Recognition

Animal individual identification is a technology that enables precise distinction and identity confirmation of different individuals by analyzing unique features—such as facial characteristics, snout patterns, spots, or markings—or specific organs. Its core components include feature extraction (e.g., facial features, spot patterns), feature matching (comparing extracted features with database records), and identity confirmation (verifying individual identities). Ultimately, this process predicts the location and species of animals in images. With the rapid development of YOLO-series algorithms, an increasing number of researchers have integrated YOLO with animal individual identification.

In practical applications, animal individual identification faces challenges such as model deployment difficulties, illumination variations, pose diversity, and occlusion. However, YOLO algorithms demonstrate strong robustness and real-time processing capabilities, enabling the stable detection of target regions in complex environments. Combined with data augmentation and transfer learning, they enhance model generalization.

To mitigate deployment difficulties in resource-constrained edge devices, the literature [28,31,43,56] employed strategies such as model pruning, quantization, knowledge distillation, lightweight module integration, and backbone network replacement. These approaches significantly reduced computational complexity and parameter counts while maintaining performance. For example, the literature [31] replaced the YOLOv7 backbone with EMobileNet, achieving a compact model with only 0.97 M parameters, a mean accuracy of 99.34%, and a frame rate of 120 FPS. In practical scenarios, challenges such as motion blur, severe occlusions, and low image quality are inevitable. To enhance model robustness under these conditions, the literature [17,53,54] combined data augmentation techniques (e.g., random cropping, rotation, brightness adjustment) with attention mechanisms (e.g., CBAM, SE, CA modules). These methods improved feature extraction and prioritization in degraded inputs. Notably, the literature [53] applied data augmentation to swine datasets and integrated attention mechanisms with optimized neck feature fusion in YOLOv5, achieving a swine individual identification accuracy of 98.4%.

In practical animal farming, YOLO-series algorithms have achieved efficient, accurate, and low-cost solutions for individual identification through various algorithm optimizations. These methods provide reliable technical support for livestock management and wildlife monitoring, significantly improving operational efficiency and animal welfare standards.

#### 3.1.3. Behavior Recognition and Monitoring

Animal behavior recognition and monitoring is a technology that enables the continuous identification of normal and abnormal behaviors by analyzing animal movements, postures, and activity patterns. This approach provides real-time insights into health status and welfare levels, allowing farmers or researchers to promptly detect anomalies such as disease, stress, or injury, thereby facilitating early intervention to mitigate economic losses and enhance animal welfare.

In this field, researchers increasingly leverage the efficient object detection capabilities of YOLO-series algorithms to localize animals in video streams, followed by keypoint detection or pose estimation to extract motion features for behavior analysis. These advancements not only improve farm productivity and economic outcomes but also modernize husbandry practices by reducing labor demands.

The literature [40,41,58,59] has demonstrated that optimized YOLO variants achieve mean recognition accuracies exceeding 95% for livestock datasets. For instance, integrating GCNet and Swin Transformer into YOLOv5’s backbone, BiFPN into its neck, and Coordinate Attention into its head resulted in 99.5% accuracy and 156.3 FPS for dairy cow behavior recognition [58]. Similarly, the literature [59] enhanced YOLOv7 with hybrid attention–convolution modules and optimized spatial pyramid structures, achieving 97.3% accuracy with reduced parameters. The literature [40,41] further refined YOLOv8 by modifying convolutional layers and incorporating attention mechanisms, attaining 96.5% accuracy for beef cattle behavior analysis.

This technology holds critical value in disease early warning. Real-time behavioral analysis enables the early detection of health issues, supporting preventive management. For ducks, the literature [32] introduced SENet attention mechanisms and WIoU v3 loss functions, achieving 94.4% accuracy in low-light conditions while reducing the model size by 2.8 MB and parameters by 8.7%. For fish, the literature [60] integrated RFB modules and CBAM attention into YOLOv5, optimizing its FPN to achieve 99.5% recognition accuracy, though dataset diversity remains a limitation. For chickens, the literature [61,62,63] improved YOLOv5’s performance via attention mechanisms, dataset augmentation, and spatial pyramid optimizations, reaching 99.6% accuracy, yet challenges persist in small-target detection and occluded scenarios. For sheep, the literature [66,67,68] established behavior recognition systems through dataset curation, model lightweighting, and inference strategies, offering tools for disease prevention and ethological research, though further optimizations in model size and accuracy are needed.

#### 3.1.4. Biomass Estimation

Biomass estimation is a technology that predicts total population size or individual growth status by accurately identifying animal counts, organ characteristics, or trunk features. It provides critical data for optimizing resource allocation, formulating scientific breeding plans, and assessing ecosystem health in aquaculture and ecological research. Integrating YOLO algorithms with biomass estimation enables automated population counting and growth monitoring in complex environments, forming a foundation for intelligent farming and animal welfare management.

For sheep, the literature [69] incorporated a bidirectional crossing-line counting method into YOLOv5, improving counting accuracy while reducing labor and resource costs. In fish biomass estimation, the literature [70] applied channel pruning and model lightweighting to YOLOv5, achieving over 90% mean accuracy at 13 FPS, though further improvements in model efficiency are needed. For shrimp, the literature [71,72] enhanced YOLO-based frameworks with attention mechanisms and adaptive frame-skipping strategies, achieving 82.57% counting accuracy and 97.47 FPS on edge devices [72]. Poultry-focused research [73] addressed challenges like uneven lighting and occlusion in chicken coops by integrating depthwise convolution, attention mechanisms, and optimized activation functions into YOLOv7, achieving over 96% counting accuracy. For ducks, the literature [74] utilized dual datasets (body and head) and enhanced YOLOv7’s backbone with attention mechanisms, achieving 97.57% accuracy, though performance in complex scenarios requires further refinement. In cattle monitoring, the literature [19,75,76] integrated transfer learning, data augmentation, and attention mechanisms into YOLO variants, achieving over 92% accuracy for individual recognition, yet advancements in dataset diversity and sensor technology remain essential. For swine, the literature [77,78] optimized YOLO architectures with attention mechanisms and automated counting algorithms, attaining 96.58% and 98.11% counting accuracy at 22 FPS and 10 FPS, respectively, meeting practical deployment requirements.

#### 3.1.5. Analysis of Phenotypic Application Differences in Animals

As demonstrated in Table 6, animal phenotyping data have become a cornerstone of modern intelligent livestock farming, being extensively utilized in applications such as body size estimation, individual identification, behavioral pattern monitoring, and biomass prediction. However, traditional phenotyping methodologies face significant limitations: manual recognition relies on labor-intensive manual measurements and visual inspections, suffering from low efficiency, subjective bias, and scalability challenges. While traditional deep learning frameworks like Faster R-CNN [79] and Mask R-CNN [16] partially enhance automation, their two-stage detection pipelines introduce high computational complexity, poor real-time performance, and fragile robustness under complex conditions (e.g., uneven illumination, dense target clustering, severe occlusion), which makes it difficult to meet the needs of dynamic farming. These methods also exhibit excessive hardware dependency and high deployment costs, further restricting their applicability in resource-constrained environments such as field monitoring and edge devices.

To address these challenges, the YOLO (You Only Look Once)-series algorithms provide efficient solutions for animal phenotyping through their innovative architectures and technical characteristics. Their core advantages are manifested in three dimensions, as follows:

(1) Non-invasive identification: Achieved through non-contact image or video analysis and avoiding physical interference with animals (e.g., stress-induced responses from radio-frequency tagging), thereby ensuring animal welfare and health.
(2) Cross-species generalization: Enabled by multi-scale feature fusion and adaptive data augmentation, YOLO algorithms adapt to diverse phenotyping needs across aquatic animals (e.g., fish), terrestrial livestock (e.g., cattle, sheep), and avian species (e.g., chickens, ducks).
(3) High robustness in complex scenarios: Enhanced through attention mechanisms, Feature Pyramid Networks, and dynamic data augmentation, YOLO maintains superior detection accuracy under challenging conditions such as low illumination, dense occlusion, and small-target detection.

To further validate YOLO’s differentiated performance in intra-species phenotyping analysis, we examined cattle herd management in complex environments.

**Figure 5:**
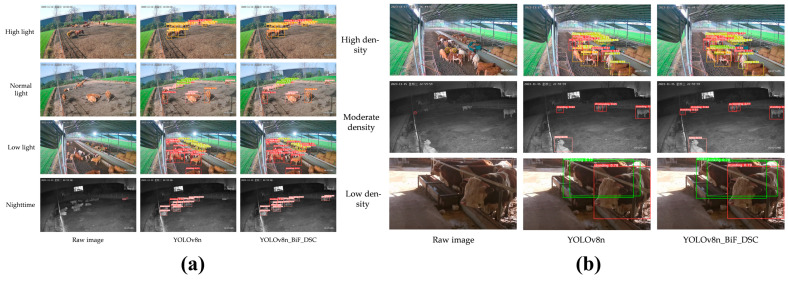
Comparison of YOLO algorithm recognition performance in complex scenarios. (**a**) depicts different lighting environments; (**b**) illustrates varying degrees of density).

(1) Comparative Analysis of Cattle Posture Recognition Algorithms. As shown in Table 8, the literature [40] demonstrates that YOLO-based algorithms exhibit superior performance compared to two-stage frameworks like Faster R-CNN in cattle posture recognition, achieving significant improvements in accuracy, recall rate, mAP50, and mAP50:95. This further validates the feasibility of YOLO-based solutions for cattle posture recognition. Notably, among YOLO variants—including YOLOX, YOLOv7, YOLOv8, and YOLOv8n_BiF_DSC—performance metrics such as accuracy, recall rate, mAP50, and mAP50:95 show gradual increments. While subtle differences exist across YOLO family algorithms under identical scenarios, their overall recognition rates remain consistently high. Specifically, YOLOv8n_BiF_DSC achieves an mAP50 of 96.5%, meeting practical requirements for intelligent cattle health monitoring and sustainable farming.

(2) Comparative Analysis of YOLO-Series Algorithms in Complex Scenarios. The literature [40] compared the performance of YOLO-based algorithms under varying lighting conditions (Figure 5a) and crowd densities (Figure 5b) for cattle posture recognition. Under normal lighting and low-density scenarios, both YOLOv8n and the improved YOLOv8n_BiF_DSC algorithm demonstrated excellent recognition accuracy, exhibiting minimal false detections and omissions. However, in complex environments such as low-light, high-light, or densely packed scenes, image noise increased, and critical features were severely degraded. While YOLOv8n could still identify most cattle postures, minor errors in detection and omission persisted. In contrast, the enhanced YOLOv8n_BiF_DSC algorithm maintained excellent recognition accuracy under these challenging conditions, proving the robustness of YOLO-series algorithms in adverse environments. For instance, in extremely dark conditions, attention mechanisms and convolutional optimizations within YOLO frameworks enable reliable animal phenotyping recognition, providing technical support for intelligent animal identification and management.

The YOLO-series algorithms have emerged as one of the most competitive solutions in intelligent animal phenotyping through continuous technological iterations and scenario-specific optimizations. Looking ahead, with the integration of lightweight design, multimodal fusion, and self-supervised learning, their application potential in highly dynamic and complex environments will be further unleashed, providing sustained technological impetus for precision livestock farming and animal science research.

## 4. Discussion

### 4.1. Future Development Directions

**Figure 6:**
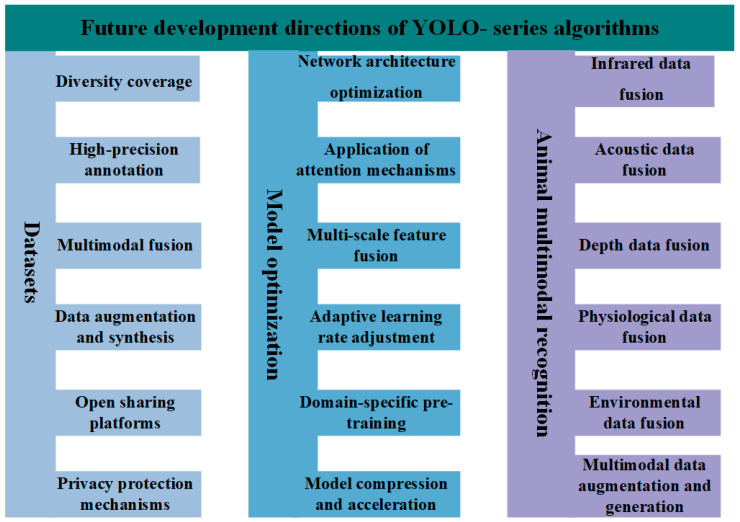
Diagram of the future development directions of YOLO-series algorithms.

In the current context, object detection technology has developed rapidly and is widely applied. However, its existing detection and perception capabilities still fall short of fully meeting the extensive application demands and industry requirements. Therefore, there is a need for more advanced YOLO object detection models and optimization strategies. Based on the current research and application status of YOLO object detection models, the following issues are summarized, and research prospects are proposed (refer to Figure 6 for details).

#### 4.1.1. Datasets

The development of animal phenotype datasets is the core driving force behind the advancement of intelligent farming. Currently, dataset construction is evolving towards greater diversity coverage, high-precision annotation, multimodal fusion, data augmentation and synthesis, and open sharing.

Firstly, datasets need to encompass a wider range of species (such as livestock, poultry, aquatic animals, and wildlife), diverse farming environments (indoor, outdoor, aquatic), and data across all growth stages to enhance the generalization capabilities of models. Secondly, through refined annotation (keypoints, bounding boxes, semantic segmentation) and standardized protocols combined with automated annotation tools, the quality and comparability of data can be ensured. Simultaneously, integrating multimodal data such as visual, infrared, and acoustic information can comprehensively analyze animal behavior, physiological states, and environmental interactions. Moreover, synthetic data technologies based on Generative Adversarial Networks (GANs) [80] and Diffusion Models (DDPMs) [81] can effectively address the scarcity of real data. Additionally, promoting the development of open data platforms while considering animal welfare and privacy protection (e.g., non-invasive collection, data anonymization) is crucial for ensuring data ethics and compliant applications. These advancements provide a solid foundation for animal phenotype research, enabling precise health monitoring (disease early warning, nutritional assessment), behavioral welfare optimization (movement, social analysis), efficient breeding management (phenotypic screening, genetic improvement), and sustainable resource utilization (precision feeding, environmental regulation) in intelligent farming. Ultimately, these efforts aim to improve farming efficiency, ensure animal welfare, and achieve ecological sustainability. In the future, with the iteration of data technologies and cross-domain collaboration, animal phenotype datasets will continue to propel agricultural intelligence towards higher levels of precision and humanization.

#### 4.1.2. Model Optimization

YOLO-series algorithms have been widely adopted in animal phenotype applications, yet there remains significant potential for enhancement under the rapid evolution of future technologies. The YOLO family of algorithms continues to exhibit opportunities for refinement in network architecture optimization, attention mechanism integration, multi-scale feature fusion, adaptive learning rate adjustment, domain-specific pretraining, and model compression/acceleration.

The YOLO model architecture can be optimized by adjusting parameters such as network depth, width, and convolutional kernel sizes to improve feature propagation efficiency. This enables the model to better capture subtle differences in animal phenotypes, which is critical for distinguishing species with similar appearances but divergent behavioral patterns.

Attention mechanisms enable the model to automatically focus on the most important parts of an image during processing. These include Spatial Attention [82] and Channel Attention [83], which help the model ignore background noise and concentrate on the key features of the target object. In complex natural environments, animals often blend into their surroundings. Using attention mechanisms can enable the model to more accurately locate and identify the animals of interest.

Multi-scale feature fusion involves combining feature maps from different levels to obtain information that includes both global context and local details. This process typically involves the use of Feature Pyramid Networks (FPNs) [84] or variations of the U-Net [85] architecture. This not only improves detection accuracy but also enhances the ability to recognize animals of different sizes and distances, making it suitable for various scenarios, from close-range observation to remote monitoring.

Employing adaptive learning rate adjustment strategies [86] allows the learning rate to be dynamically adjusted based on changes in the loss function during training, ensuring rapid convergence and avoiding local minima. When dealing with large-scale datasets, adaptive learning rate adjustment can significantly speed up the training process and reduce the risk of overfitting. This is particularly valuable for research that requires frequent model updates to address emerging animal populations or changing ecological environments.

Pretraining models on specific types of animals and utilizing transfer learning methods [87] to apply existing knowledge to new tasks can fine-tune models to optimal performance with only a small amount of annotated data. This approach greatly reduces the need for large amounts of annotated data and lowers labor costs. This strategy is especially useful for research areas where obtaining large amounts of high-quality annotated data is difficult, such as rare species conservation or exploration of unique ecosystems.

Since animal farming requires practical deployment, it is crucial to research methods such as pruning and quantization [88] to adapt models for deployment on resource-constrained devices without sacrificing too much accuracy.

#### 4.1.3. Animal Multimodal Recognition

The YOLO-series algorithms in animal multimodal recognition involve integrating visual, acoustic, infrared, depth, and other data sources to comprehensively analyze animal phenotypic characteristics (e.g., morphology, behavior, physiological states). As a leading real-time object detection technology, the YOLO series holds broad prospects in multimodal recognition. Its developmental directions include infrared data fusion, acoustic data fusion, depth data fusion, physiological data fusion, environmental data fusion, and multimodal data augmentation and generation, among others.

Infrared Data Fusion [89,90,91]: By integrating YOLO algorithms with infrared cameras and combining thermal imaging with visible light, animal body temperature distribution can be detected. Alternatively, specialized multi-branch networks can be designed to extract features from visible and infrared images separately, followed by information integration through feature fusion modules (e.g., weighted summation, attention mechanisms).

Acoustic Data Fusion [92,93,94]: Associating YOLO-derived visual data with spectrograms or deep learning models (e.g., CNNs or RNNs) for acoustic feature extraction enables the recognition of animal emotional states (e.g., stress, estrus) or behavioral patterns (e.g., feeding, aggression).

Depth Data Fusion [95,96,97]: Utilizing depth cameras to acquire 3D point cloud data of animals combined with the YOLO-based detection of keypoints (e.g., joints, head) facilitates precise pose estimation and body condition scoring, which is applicable to scientific growth monitoring or breeding evaluation.

Physiological Data Fusion [98,99,100]: Integrating YOLO algorithms with wearable devices (e.g., heart rate monitors, accelerometers) allows the visual localization of individuals while capturing physiological metrics (e.g., heart rate, activity levels), enabling comprehensive health monitoring and assessment.

Environmental Sensor Data Integration [101,102,103]: Combining YOLO algorithms with environmental sensors (e.g., temperature–humidity sensors, gas sensors) helps analyze correlations between animal behavior and environmental factors, optimizing rearing conditions for animal welfare.

Multimodal Data Augmentation and Generation [104,105,106]: Cross-modal data augmentation techniques and generative models (e.g., Generative Adversarial Networks (GANs) or Denoising Diffusion Probabilistic Models (DDPMs)) can enhance dataset diversity and improve model generalization capabilities.

## 5. Conclusions

With the rapid development of YOLO-series algorithms, they have become a significant direction in the field of animal target detection. This paper outlines the evolution of YOLO-series algorithms and presents a comparative table highlighting the differences and similarities among various versions. It discusses the importance of datasets in target detection and analyzes the applications and improvements of YOLO algorithms across multiple domains based on recent research. Additionally, it predicts future directions for YOLO-series algorithms. Through this comprehensive review and analysis, the paper aims to provide readers with new insights and perspectives on the application of YOLO algorithms in animal phenotyping.

## Figures and Tables

**Table 1:** Mechanisms, advantages, and limitations of different YOLO versions.

Model	Machine-Processed	Superiority	Boundedness
YOLO v1	First proposed a single-stage detection framework, dividing images into grids. Each grid predicts bounding boxes and class probabilities for objects of the same category, filtered by non-maximum suppression (NMS).	Detection speed with real-time capability; improved recall rate (reduced missed detections); strong generalization and ease of extension.	Limited detection and localization accuracy, especially for small objects. Grid-based division restricts precise object localization.
YOLO v2	Introduced DarkNet-19 backbone, batch normalization, high-resolution input, anchor boxes, and K-Means clustering for bounding box optimization.	Improved detection accuracy while maintaining speed; enhanced generalization for diverse object types.	Limited adaptability in complex scenes; increased complexity in model tuning due to new parameters.
YOLO v3	Utilized Darknet-53 backbone with Feature Pyramid Network (FPN) for multi-scale prediction. Split architecture into input, backbone, neck, and detection head.	Enhanced accuracy, small-object detection, and feature extraction capabilities.	High computational resource consumption; prone to missed or false detections in dense object overlaps.
YOLO v4	Integrated Mosaic data augmentation, CBM (Convolution-BatchNorm-Mish), SAT (Self-Adversarial Training), CSPDarknet53 backbone, SPP module, and FPN + PAN for multi-scale feature fusion and receptive field expansion.	Balanced speed and accuracy with high detection performance across diverse objects.	Complex architecture; high training/deployment difficulty and computational demands.
YOLO v5	Introduced adaptive anchor calculation, focus slicing, BottleneckCSP modules, Mosaic augmentation, and PANet neck for optimized feature transfer.	Improved training/inference speed; flexible deployment with multiple model sizes.	Structural complexity requiring computational resources; may need additional optimization for specific scenarios.
YOLOX	Introduced anchor-free design, decoupled heads, SimOTA dynamic label assignment, and simplified data augmentation/end-to-end optimization.	Excels in dense target scenarios and complex environments; reduces hyperparameter tuning complexity via anchor-free architecture; enables easy deployment.	Small target detection rates require improvement; high computational costs persist.
YOLO v6	Designed an efficient decoupled-head, hybrid channel strategy to reduce redundancy and optimized loss functions and training strategies.	Enhanced efficiency with balanced speed and accuracy through architectural and training improvements.	Limited performance gains in highly complex scenes.
YOLO v7	Combined Transformer attention mechanisms with path aggregation networks, optimized convolutional layers, and deeper feature extractors.	Improved accuracy in complex scenes with better speed–accuracy trade-off; supports multi-task extensions.	High model complexity and training costs.
YOLO v8	Enhanced backbone, detection head (e.g., C2f structure for gradient flow), decoupled head, and channel adaptation for multi-scale tasks.	Versatile for detection, segmentation, and classification; easy deployment and modification (pip-installable).	Increased parameters and training costs; real-time performance needs optimization.
YOLO v9	Built on YOLOv7 with programmable gradient information and Generalized Efficient Layer Aggregation Network (GELAN) for efficient gradient path planning.	High performance with reduced parameters and computations; suitable for resource-constrained scenarios.	Inaccurate small-object detection.

**Table 2:** Experimental results of YOLO v1-2 on PASCAL VOC.

Basic Model	Model Variant	Input Size	mAP	FPS
YOLO v1	Fast YOLO	448 × 448	52.7	155
	YOLO v1	448 × 448	63.4	45
YOLO v2	YOLO v2	288 × 288	69.0	91
	YOLO v2	352 × 352	73.7	81
	YOLO v2	416 × 416	76.8	67
	YOLO v2	480 × 480	77.8	59
	YOLO v2	544 × 544	78.6	40

**Table 3:** Experimental results of YOLO algorithms (excluding YOLO v1-2) on MS COCO.

Basic Model	Model Variant	Input Size	GPU	*A* *P*	*AP*_50_ (%)	*AP*_75_ (%)	*AP*_*S*_ (%)	*AP*_*M*_ (%)	*AP*_*L*_ (%)	FPS
YOLO v3	YOLO v3	320 × 320	Tesla M40 GPU	28.2	51.5	29.7	11.9	30.6	43.4	45
	YOLO v3	416 × 416		31.0	55.3	32.3	15.2	33.2	42.8	35
	YOLO v3	608 × 608		33.0	57.9	34.4	18.3	35.4	41.9	20
	YOLO v3-SPP	608 × 608		36.2	60.6	38.2	20.6	37.4	46.1	20
YOLO v4	YOLO v4	416 × 416	Tesla M40 GPU	41.2	62.8	44.3	20.4	44.4	56.0	38
	YOLO v4	512 × 512		43.0	64.9	46.5	24.3	46.1	55.2	31
	YOLO v4	608 × 608		43.5	54.7	47.3	26.7	46.7	53.3	23
YOLO v5	YOLO v5-N	640 × 640	Tesla T4 GPU	28.0	45.7	-	-	-	-	602
	YOLO v5-S	640 × 640		37.4	56.8	-	-	-	-	376
	YOLO v5-M	640 × 640		45.4	64.1	-	-	-	-	182
	YOLO v5-L	640 × 640		49.0	67.3	-	-	-	-	113
YOLO v6	YOLO v6-N	640 × 640	Tesla T4 GPU	35.9	51.2	-	-	-	-	802
	YOLO v6-T	640 × 640		40.3	56.6	-	-	-	-	449
	YOLO v6-S	640 × 640		43.5	60.4	-	-	-	-	358
	YOLO v6-M	640 × 640		49.5	66.8	-	-	-	-	179
	YOLO v6-L-ReLU	640 × 640		51.7	69.2	-	-	-	-	113
	YOLO v6-L	640 × 640		52.5	70.0	-	-	-	-	98
YOLO v7	YOLOv7-tiny	416 × 416	V100 GPU	35.2	52.8	37.3	15.7	38.0	53.4	273
	YOLO v7	640 × 640		51.2	69.7	55.5	35.2	56.0	66.7	118
	YOLO v7-X	640 × 640		52.9	71.1	57.5	36.9	57.7	68.6	98
	YOLO v7-E6	1280 × 1280		55.9	73.5	61.1	40.6	60.3	70.0	54
	YOLO v7-D6	1280 × 1280		56.3	73.8	61.4	41.3	60.6	70.1	43
	YOLO v7-E6E	1280 × 1280		56.8	74.4	62.1	40.8	62.1	70.6	35
YOLO v8	YOLO v8n	640 × 640	V100 GPU	37.3	52.6	-	-	-	-	-
	YOLO v8s	640 × 640		44.9	61.8	-	-	-	-	-
	YOLO v8m	640 × 640		50.2	67.2	-	-	-	-	-
	YOLO v8l	640 × 640		52.9	69.8	57.5	35.3	58.3	69.8	
	YOLO v8x	640 × 640		53.9	71.0	58.7	35.7	59.3	70.7	
YOLO v9	YOLO v9-T	640 × 640	V100 GPU	38.3	53.1	41.3	-	-	-	-
	YOLO v9-S	640 × 640		46.8	63.4	50.7	26.6	56.0	64.5	-
	YOLO v9-M	640 × 640		51.4	68.1	56.1	33.6	57.0	68.0	-
	YOLO v9-C	640 × 640		53.0	70.2	57.8	36.2	58.5	69.3	-
	YOLO v9-E	640 × 640		55.6	72.8	60.6	40.2	61.0	71.4	-

**Table 4:** Common public datasets and their characteristics.

Dataset		Categories	Image Size (Input Resolution)	Year Established	Key Features	Download Address
Common Object Detection Datasets	Pascal VOC	20 classes	Variable (resized to fixed resolution for model input)	2005	Foundational dataset for object detection with 20 common object categories.	http://host.robots.ox.ac.uk/pascal/VOC/voc2007/index.html (accessed on 20 January 2025)
	ImageNet	9000 classes	Variable	2010	Large-scale dataset with extensive image classification labels.	https://image-net.org/download.php (accessed on 20 January 2025)
	COCO	80 classes	Typically resized to 608 × 608 or model-specific resolutions	2014	Rich contextual information; complex real-world scenes with precise annotations.	https://cocodataset.org/ (accessed on 20 January 2025)
	Google Open Images	600 classes	Variable	2017	Large-scale dataset and multi-object scenes.	https://storage.googleapis.com/openimages/web/download_v7.html (accessed on 20 January 2025)
Animal Phenotyping Datasets	AP-10 K Animal Pose Estimation [33]	54 classes	Variable	2021	Benchmark for animal pose estimation with diverse animal postures.	https://github.com/AlexTheBad/AP10K (accessed on 20 January 2025)
	Sheep Grazing Dataset [34]	5 classes	Variable	2024	Supports intelligent sheep grazing management with behavioral analysis.	https://zenodo.org/records/11313800 (accessed on 20 January 2025)
	Cow Body Condition Scoring [35]	5 classes	1297 × 720	2024	Enables automated body condition assessment for cattle health monitoring.	https://www.scidb.cn/en/detail?dataSetId=16b8bdaf31ee4c8b9891fc7e9df6e41c (accessed on 20 January 2025)
	Salmon Health Assessment [36]	2 classes	1920 × 1080	2024	Provides data for salmon growth status evaluation and health risk assessment.	https://data.mendeley.com/datasets/rvrt4zs969/1 (accessed on 20 January 2025)

**Table 5:** Self-built datasets and their characteristics.

Custom Datasets	Total Samples	Data Augmentation Methods	Hardware	Application Scenarios
Sheep Face Dataset [37]	4000	Mosaic augmentation; random adjustments to hue, saturation, brightness; flipping, shearing, scaling, translation	Binocular cameras	Accurate detection and localization of goat faces in complex environments, supporting precision livestock farming with technical insights.
Dairy Cow Body Condition Dataset [38]	8972	Manual filtering	Hikvision network cameras	Commercial body condition scoring for dairy cows, providing theoretical foundations and intelligent solutions for modern dairy farming.
Meat Pigeon Behavior Dataset [39]	10,320	Fog simulation; random flipping; noise injection; blurring	Hikvision DS-2CD3T47EDWD-L (4 mm) cameras	Technical reference for intelligent meat pigeon breeding and scientific management, enhancing automation in poultry farming.

**Table 6:** Data set labeling method and characteristics.

Annotation Method	Significance	Application Scenarios
Bounding Box Annotation	Provides rough location information of the target object, suitable for object detection tasks.	Animal detection, quantity statistics, target tracking.
Semantic Segmentation Annotation	Provides pixel-level fine annotation, suitable for tasks that require precise target boundaries.	Animal contour extraction, background separation, scene understanding.
Instance Segmentation Annotation	Provides pixel-level annotation while distinguishing different individuals.	Analysis of multi-animal scenes, individual behavior research, phenotypic feature quantification.
Keypoint Annotation	Provides the posture and structural information of the target animal, suitable for pose estimation and behavior analysis tasks.	Animal pose estimation, behavior recognition, phenotypic feature measurement.
Trajectory Annotation	Provides the movement information of the target animal, suitable for behavior analysis and tracking tasks.	Animal behavior analysis, target tracking, group behavior research.

**Table 7:** Application results of YOLO-series algorithm in animal phenotypes.

Scenario	Animal	Algorithm	Improvement Method	Performance Indicator	Reference
Body Size Estimation	Cow	YOLOv5	Combined with Siamese network	mAP50: 95.13%	[42]
	Cow	YOLOv5	Sparse BN layer, channel pruning	Model size reduced by 86.10%, parameter quantity decreased by 88.19%, and FLOPs reduced by 63.25%, respectively	[43]
	Cow	YOLOv5	Attention mechanism of bilateral filtering, optimized pooling	mAP50: 90.74%	[44]
	Pig	YOLOv5	Established a body weight prediction system	The prediction error meets the breeding requirements	[45]
	Pig	YOLOv5	Introduced MobilenetV3 network and attention mechanism	The model is reduced to 10.2 M, and the error rate is lower than 2%	[29]
	Chicken	YOLOv8	Introduced volume and mass calculation system	The prediction error rate is lower than 5%	[46]
	Cow	YOLOv8	Established a method for estimating cow body weight	P: 97.8%, R: 96.4%, mAP50: 99.0%	[47]
	Cow	YOLOv3	Mean filtering algorithm, custom FilterLayer layer	P: 99.18%, R: 97.51%, mAP50: 99.0%, frame rate: 21 FPS	[48]
	Sheep	YOLOv7	Body length estimation based on distance	The error rate is low and meets the actual needs	[49]
	Sheep	YOLOv8	Combined with Roboflow algorithm	mAP50: 88.2%	[50]
	Pig	YOLOv5	Combined with U-Net network	mAP50: 92%	[51]
Individual Recognition	Cow	YOLOv3	Integrated individual recognition and detection system	mAP50: 96%	[27]
	Cow	YOLOv8	Introduced hash algorithm and sliding window for segmentation	mAP50: 98.6%	[52]
	Pig	YOLOv5	Introduced attention mechanism, optimized neck feature fusion	mAP50: 98.4%	[53]
	Pig	YOLOv8	LSKA attention mechanism, optimized downsampling, loss function	mAP50: 94.76%, frame rate: 79 FPS	[54]
	Pig	YOLOv7	EMobileNet backbone network, Horizontal–Vertical Attention Mechanism (HVAM)	Model parameters are 0.97 M, mAP50: 99.34%, frame rate: 120 FPS	[31]
	Sheep	YOLOv4	Introduced GhostNet module, improved feature fusion network	mAP50: 96.7%, frame rate: 28 FPS	[28]
	Sheep	YOLOv5	CBAM attention mechanism	P: 97%, R: 89%, mAP50: 93.5%, frame rate: 140 FPS, model parameters are 14.68 M	[17]
	Sheep	YOLOv5	ShuffleNetv2 module and Ghost module	mAP50: 97.8%, model parameters are 9.5 M.	[55]
	Fish	YOLOv2	Continuous frame optimization classification method	The mAP50 of the two data sets was 93.94% and 97.06%, respectively	[26]
	Fish	YOLOv3	Introduced MobileNet network	Both the parameter quantity and the mean average precision are improved compared with the baseline model	[56]
	Fish	YOLOv7	Improved convolution kernel, detection head, network pruning	Average accuracy rate: 92.86%; the amount of calculation is reduced by about 35%	[57]
Behavior Recognition and Monitoring	Dairy Cow	YOLOv5	Introduced GCNet and Swin Transformer in the backbone network; introduced BiFPN in the neck; introduced CA (Coordinate Attention) attention mechanism in the head.	P: 99.7%, R: 99.5%, mAP50: 99.5%, frame rate: 156.3 FPS	[58]
	Dairy Cow	YOLOv7	Added self-attention and convolution hybrid module (ACmix), improved the downstream task of ByteTrack	P: 97.3%, R: 96%, mAP50: 97.3%	[59]
	Cow	YOLOv8	Optimized the conv convolution layer, introduced the attention mechanism	P: 93.6%, R: 92.9%, mAP50: 96.5%	[40,41]
	Duck	YOLOv8	Introduced SENet attention mechanism, WIoU v3 loss function	mAP50: 94.4%, the model is reduced by 2.8 MB, and the parameter quantity is reduced by 8.7%	[32]
	Fish	YOLOv5	RFB module, CBAM attention mechanism, optimized FPN module	P: 99.8%, R: 99.5%, mAP50: 99.5%	[60]
	Chicken	YOLOv5	Attention mechanism, optimized spatial pyramid pooling module	P: 93.6%, R: 99.5%, mAP50: 95.45%	[61]
	Chicken	YOLOv5	Dataset and model optimization	P: 93.7%, R: 84.6%, mAP50: 90.9%	[62]
	Chicken	YOLOv5	Loss function	P: 99.9%, R: 99.2%, mAP50: 99.6%	[63]
	Pigeon	YOLOv4	Introduced GhostNet	mAP50: 97.06%, frame rate: 35.71 FPS	[64]
	Pig	YOLOv8	Introduced Multi-Path Coordinate Attention (MPCA) mechanism, optimized C2f	P: 88.2%, R: 92.2%, mAP50: 95.3%	[65]
	Sheep	YOLOv8	Model lightweight, introduced attention mechanism, loss function	mAP50: 98.11%	[66]
	Sheep	YOLOv4	Introduced behavior reasoning strategy	mAP50: 96%, and the frame rate is 17 FPS.	[67]
	Sheep	YOLOv8	CBAM attention mechanism, improved convolution module	The mAP50 is higher than 96%, the model volume is reduced by 13.3%, the computation amount is decreased by 12.1%, and the frame rate is 52.1 FPS.	[68]
Biomass Estimation	Sheep	YOLOv5	Bidirectional line-crossing counting method	High accuracy rate, in line with practical applications	[69]
	Fish	YOLOv5	Channel pruning, model lightweight	When pruning 15%, the average accuracy rate of the model is above 90%, and the frame rate is 13 FPS	[70]
	Shrimp	YOLOv5	CBAM attention mechanism	P: 97.2%, R: 96.5%, mAP50: 96.3%	[71]
	Shrimp	YOLOv8	Adaptive frame skipping	Counting rate: 82.57%, frame rate: 97.47 FPS	[72]
	Chicken	YOLOv7	Introduced deep convolution, attention mechanism	The mAP50 is 96.9%, and the model volume is reduced to 5.6 MB	[73]
	Duck	YOLOv7	CBAM attention mechanism	P: 96.84%, R: 94.57%, mAP50: 98.72%	[74]
	Cow	YOLOv5	Introduced SPPFCSPC, GSConv, CA attention mechanism	P: 95.5%, mAP50: 95.2%, frame rate: 88 FPS	[75]
	Cow	YOLOv7	Introduced PConv, DioU, DyHead	P: 98.8%, R: 99%, mAP50: 92.1%	[76]
	Cow	YOLOv8	Transfer learning, data augmentation	P: 91%, R:83.4%, mAP50: 88.8%	[19]
	Pig	YOLOv7	Introduced REPConv, CA attention mechanism	mAP50: 96.58%, frame rate: 22 FPS	[77]
	Pig	YOLOv4	Proposed an automatic counting algorithm	mAP50: 98.11%, frame rate is approximately 10 FPS	[78]

**Table 8:** Cattle pose recognition results of different algorithms.

Model	P	R	mAP50	mAP50:95
Faster R-CNN	0.862	0.843	0.879	0.605
YOLOX	0.869	0.859	0.901	0.639
YOLOv7	0.87	0.862	0.911	0.642
YOLOv8n	0.883	0.866	0.913	0.644
YOLOv8n_BiF_DSC	0.936	0.929	0.965	0.715

## Data Availability

The data presented in this study are available on request from the corresponding author.
